# Pparα knockout in mice increases the Th17 development by facilitating the IKKα/RORγt and IKKα/Foxp3 complexes

**DOI:** 10.1038/s42003-023-05104-6

**Published:** 2023-07-14

**Authors:** Ping Wei, Wei Kou, Juan Fu, Zuojia Chen, Fan Pan

**Affiliations:** 1 Department of Otolaryngology, Ministry of Education Key Laboratory of Child Development and Disorders, National Clinical Research Center for Child Health and Disorders (Chongqing), China International Science and Technology Cooperation base of Child Development and Critical Disorders, Children’s Hospital of Chongqing Medical University, Chongqing, China; 2 Shenzhen Institute of Advanced Technology (SIAT), Chinese Academy of Sciences (CAS), 1068 Xueyuan Avenue, Shenzhen, 518055 PR China; 3 Department of Oncology, Sidney Kimmel Comprehensive Cancer Center, Johns Hopkins University School of Medicine, Baltimore, MD USA; 4 Experimental Immunology Branch, National Cancer Institute, NIH, Bethesda, MD USA

**Keywords:** Immunology, T cells

## Abstract

The helper CD4^+^ T cell-type 17 (Th17) cells and regulatory CD4^+^ T cells (Tregs) are balanced through numerous molecular regulators, particularly metabolic factors, and their alteration causes immune dysregulation. Herein, we report that peroxisome proliferator of activated receptor-alpha (*Pparα*), a lipid metabolism regulator, suppresses Th17 differentiation. We demonstrated that Pparα ablation improves Th17 and pro-Th17 factor *HIF-1α* by enhancing the expression and nuclear localization of *NFκB*-activator IκB kinase-alpha (*IKKα*). Unexpectedly, we found that IKKα directly interacts with RORγt and enhances the expression of *Il17a* gene. Meanwhile, IKKα also interacts with Foxp3, leading to the post-translational regulation of Foxp3 by elevating its proteasomal degradation, and influencing Th17 development. *Pparα* deficiency leads to enhanced Th17 development in vivo and is associated with enhanced pathology in a murine experimental autoimmune encephalomyelitis (EAE) model. Overall, our data indicate that *Pparα* may serve as a potential therapeutic target for autoimmune and inflammatory diseases.

## Abstract (web summary)

Upregulation of Pparα in activated CD4^+^ T cells inhibits IKKα expression and IKKαdependent degradation of Foxp3 and elevation of IL17a, thus could constitute a potential therapeutic target for autoimmune, cancer and inflammatory diseases

## Introduction

Naïve CD4^+^ T cells develop into highly specialized T helper (Th) subsets when activated in the presence of lineage-associated cytokines^1^. Among these Th subsets, Th17 cells lineage is characterized by the expression of the transcription factor retinoic-acid related orphan receptor-γt (RORγt) and effector cytokines interleukin-17A (IL-17A) and IL-17F^2^. Meanwhile, Tregs are characterized by the expression of the transcription factor, forkhead-box P3 (Foxp3), and suppressive capacity of immune responses^3^. While performing the opposing functions, both Th17 and iTreg lineages share elements of their differentiation pathways, including the cytokine TGF-β and the initial expression of Foxp3 in the early stages of development^4–6^. Understanding the molecular mechanisms responsible for differentiating T cells towards one or the other fate is crucial as these mechanisms are highly associated with immune dysregulation and the onset of multiple diseases, including cancer and autoimmune diseases.

A growing body of work has revealed that, in addition to cytokines, metabolic factors also play an important part in determining the fate of naïve CD4^+^ T cells upon differentiation^7,8^. For instance, the ability of naïve T cells to uptake and utilize glucose is critical for the transition from naïve T cell precursors to effector T cells which are capable of rapid growth and production of effector molecules^7,9^. Similarly, molecular mechanism underlying the glucose uptake in T cells indicates that the expression level of Glut1, a plasma membrane glucose transporter in hematopoietic cells, is at lower levels in naïve T cells and is enhanced upon activation of T cells^10,11^. This increased expression of Glut1 and glucose uptake in activated T cells depends on activating the PI3K/Akt signaling pathway. Meanwhile, activation of PI3K–Akt pathway also triggers the mTOR, the mechanistic target of rapamycin, to facilitate the glycolytic metabolism for cell proliferation and growth^7,12^.

Similarly, HIF-1α, a transcription factor responding to hypoxia, regulates glycolysis to influence glucose uptake^13,14^. It has been investigated that mTOR regulates the HIF-1α to mediate the T cell metabolism. Likewise, the absence of HIF-1α in CD8 and Th17 cells mitigates the expression of metabolic genes, including Glut1^14–17^. Chemical or genetic disruption of glucose metabolism and their associated pathways converts newly activated T cells from pro-inflammatory effector T cell fates (Th1, Th2, Th17) to an induced (i)Treg-fate^18–20^, which preferentially rely on mitochondrial respiration and lipid oxidation to meet their distinct metabolic needs^18,20,21^. Tregs have been identified to rely on mitochondrial lipid oxidation for their growth and expansion primarily due to the activation of AMP-activated kinase (AMPK), which is critical for metabolic processes and exerts opposing effects to that of mTOR^20^. Meanwhile, AMPK regulates T cell immunity by serving important roles in regulating fatty acid oxidation (FAO) and inhibiting de novo fatty acid (FA) production^22^. Peroxisome proliferator of activated receptor (*Ppar*) encompasses an important family of molecules that consists *Pparα*, *Pparg*, and *Pparδ,* and is known to play various roles in lipid metabolism, FAO, and immune regulation^23^. Previous studies found that PPARα agonist selectively suppresses the Th17 development during the progression of autoimmune diseases^24,25^. Similarly, Pparα agonist can ameliorate the severity of EAE in mice by suppressing Th1 and Th17 development^26^. Pparα was also recently found critical for a specialized subpopulation of adipose tissue-resident Tregs with a remarkable influence on metabolism^27^. Despite the apparent importance of *Pparα* across various immune cells, the role played by these enzymes in T cell differentiation is incompletely understood.

Among PPAR family, *Pparα*, a well-known fatty acid modulator, regulates the T cell immune responses, including the onset of T cell-mediated autoimmune diseases in a gender-specific manner^24,28,29^. Additionally, the role of *Pparα* has been suggested as an anti-inflammatory mediator in several immune cell types^24,30–33^. However, the precise contribution of *Pparα* in the differentiation of naïve CD4^+^ T cells to specific T effector lineage with pro- or anti-inflammatory potential remains unknown. Therefore, suspecting the rigorous role of *Pparα* in determining the effector T cell fate, we designed this study to investigate the mechanism underlying the PPARα-dependent differentiation of naïve CD4^+^ T cells to specific T effector cells. Briefly, we investigated that the upregulation of *Pparα* in activated CD4^+^ T cells inhibits the expression of *IKKα*. Meanwhile, pharmacological inhibition of *Pparα* leads to the IKKα-dependent degradation of Foxp3 and elevation of *IL17a* in an IKKα/RORγt-dependent manner.

## Results

### Genetic ablation of *Pparα* triggers Th17 development

**Fig. 1:**
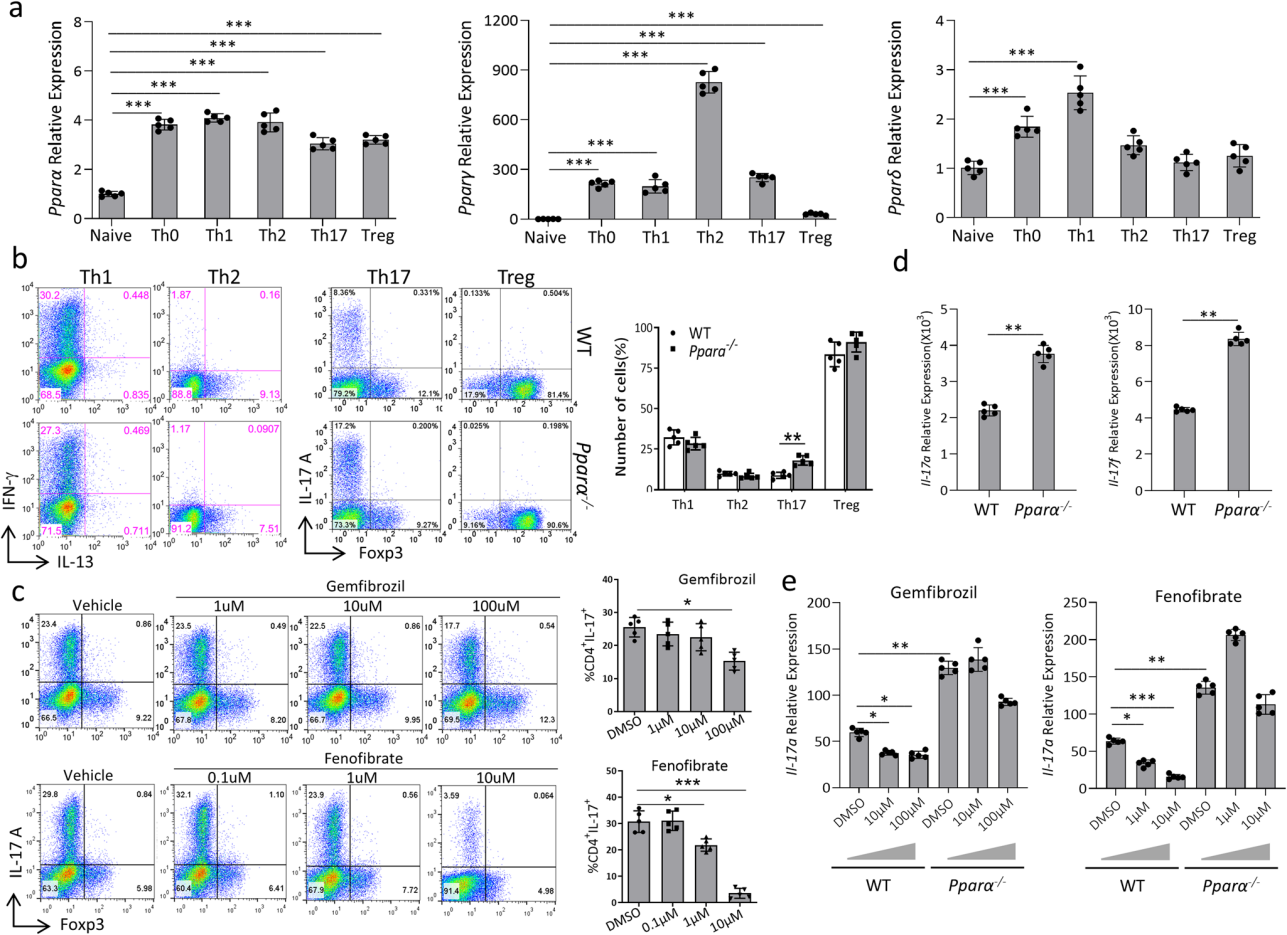
Knock-out of *Pparα* enhances the Th17 development from naïve CD4 + T cells. **a** Differential transcript levels of PPAR subtypes in different Th subsets. *Ppara* (left) *Pparg* (middle) *Pparδ* (right) expression after 24 h of skewing of naïve CD4 + T cells (CD4^+^ CD25^-^ CD62L^hi^) under Th0, Th1, Th2, Th17 and Treg polarizing conditions. **b** Representative flow-plots of IFN-γ, IL-13, IL-17A, and Foxp3 expression in naïve CD4^+^ T cells isolated from wild-type (WT) and *Ppara* knock-out (KO) mice polarized for 72 h. Naïve CD4^+^ T cells from each group were cultured under different polarizing conditions as described in the method section. **c** Representative flow plots of IL-17A and Foxp3 of CD4^+^ T cells after 72 h of Th17 polarization under varying concentrations of gemfibrozil (top) and fenofibrate (bottom). **d** *Il17a* (left) and *Il17f* (right) transcript levels after 72 h of Th17 polarization of WT and *Pparα* KO CD4^+^ T cells. **e** CD4^+^ T cells from WT and *Pparα* KO mice were cultured under Th17 polarizing conditions for 24 h along with varying concentrations of gemfibrozil (left) and fenofibrate (right), and *Il17a* transcript levels were assessed by qRT-PCR. Results are shown as the means ± SEM representative of five independent experiments. Unpaired student t-test were used for statistical analysis representing *P < 0.05, **P < 0.01, and ***P < 0.001.

In line with the potential role of PPARα in T cell fate determination, we first investigated the expression of *Pparα* in different subsets of CD4^+^ Th cells. Our results indicated that the expression of *Pparα* was up-regulated in different CD4^+^ Th cells  upon αCD3/αCD28-dependent activation of naïve CD4^+^ T cells (Fig. 1a). To further validate the effect of *Pparα* on the population of immune cell subsets, we collected lymphocytes from the spleen and thymus, and evaluated the population of different immune cells. It was determined that deficiency of *Pparα* didn’t change the population of CD11b^+^ Gr1^+^ myeloid-derived suppressor cells, CD11b^+^ CD11c^+^ dendritic cells, CD19^+^ B cells, CD8^+^ T cells and NK1.1^+^ natural killer cells (Supplementary Fig. 1a, b). We then investigated the effects of *Pparα* deficiency on naïve T cell proliferation and acquisition of effector cytokine expression. Naïve CD4^+^ T cells from WT and *Pparα* KO mice cultured under non-polarizing (Th0) conditions showed comparable proliferation by CFSE dilution (Supplementary Fig. 1c). Interferon-γ (IFN-γ) expression by these WT and *Pparα* KO cells was also comparable, while IL-2 production did appear enhanced in the absence of *Pparα* (Supplementary Fig. 1c). To further assess the effects of *Pparα* on Th differentiation, we activated naïve CD4^+^ T cells from WT and *Pparα* KO mice under different Th subset-generating conditions in vitro. *Pparα* KO CD4^+^ T cells displayed increased IL-17A expression under the Th17 polarizing condition than WT CD4^+^ T cells. In contrast, the development of Th1 and Th2 subsets remains intact in *Pparα* KO mice (Fig. 1b). Enhanced Th17 development in CD4^+^ T cells isolated from *Pparα* KO was associated with increased transcripts levels of *Il17a* and *Il17f* (Fig. 1d). These data suggest that *Pparα* plays a specific role in dampening Th17 differentiation.

### Pharmacological activation of *Pparα* reduces the Th17 development

The activation of *Pparα* requires the binding of ligands to modulate gene transcription^34^. Known natural ligands for *Pparα* include Leukotriene B (LTB) and unsaturated fatty acids^35^. Synthetic ligands, such as fenofibrate and gemfibrozil, often used as anti-diabetic drugs to modulate triglyceride levels in diabetic patients, can activate *Pparα*^36^. Evidence indicated that fenofibrate inhibited cellular proliferation; however, gemfibrozil, which had similar effects on IL-17 up-regulation, did not affect the proliferation. To further strengthen the effect of *Pparα* on Th17 cell development, we investigated whether synthetic *Pparα* ligands can modulate the Th17 development. Naïve CD4^+^ T cells from WT mice were activated under Th17 polarizing conditions in the presence of gemfibrozil and fenofibrate. After 72 h, the flow cytometric data indicated that the level of *Il17a* expression decreased in a dose-dependent manner (Fig. 1c). However, both gemfibrozil and fenofibrate didn’t affect the expression of Foxp3 (Fig. 1c). Furthermore, *Il17a* transcript levels in developing Th17 cells from WT mice were decreased by varying concentrations of gemfibrozil and fenofibrate (Fig. 1e), demonstrating transcriptional regulation of *Il17a* by *Pparα*. As expected, *Pparα* KO Th17 cells expressed higher levels of *Il17a* compared to WT Th17 cells, and the higher level of *Il17a* expression was maintained under various doses of gemfibrozil and fenofibrate. This data suggests that pharmacological targeting of *Paprα* through gemfibrozil and fenofibrate inhibits the development of Th17 cells.

### Loss of *Pparα* assists the formation of IKKα/RORγt and IKKα/Foxp3 complexes to modulate the expression of *Il17a*

**Fig. 2:**
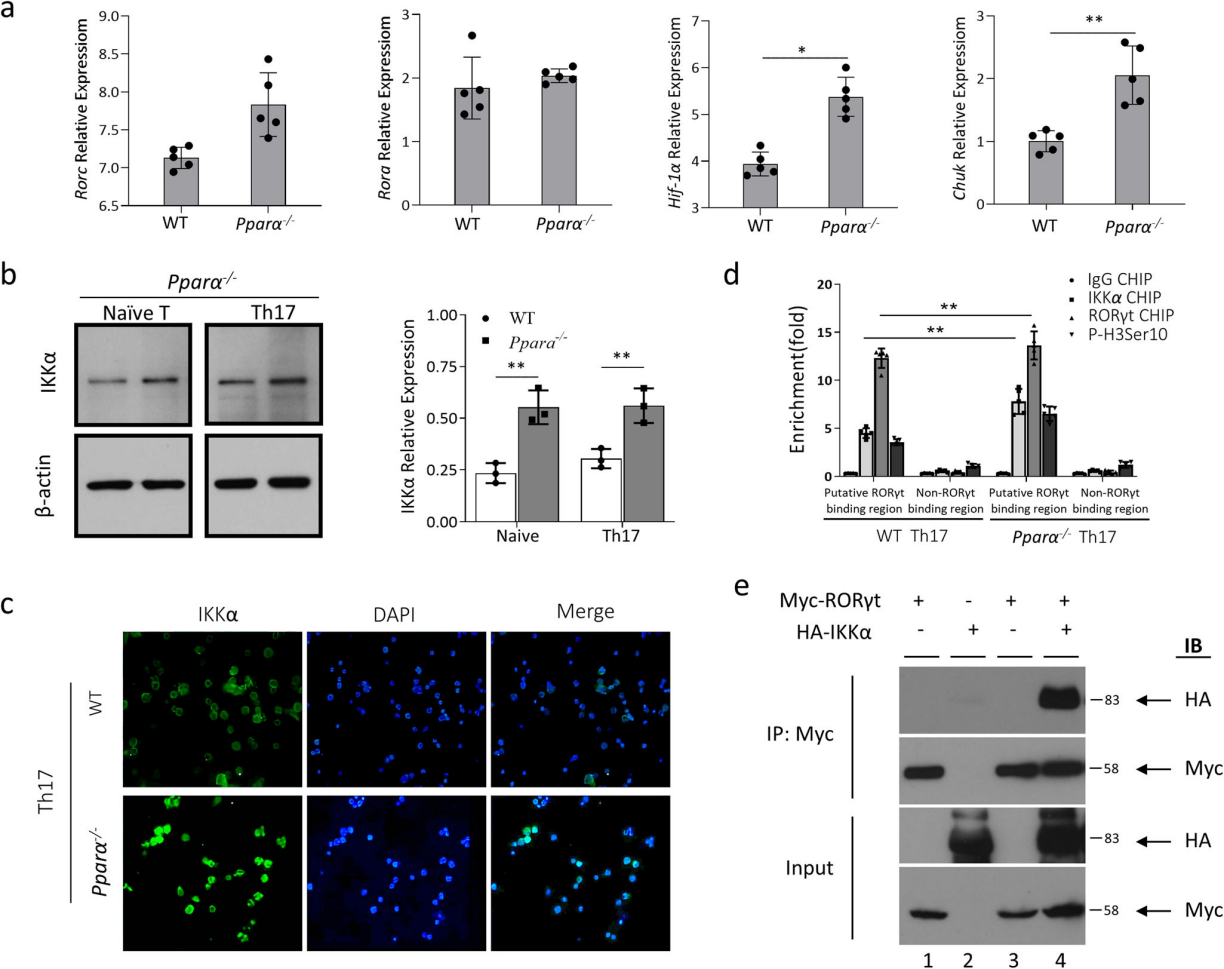
Loss of *Pparα* modulates the expression of *Il17a* through IKKα/RORγt complex. **a** Naïve CD4^+^ T cells (CD4^+^ CD25^-^ CD62L^hi^) from WT and *Pparα* KO mice were cultured under Th17 polarizing condition for 72 h, and *Rorc*, *Rora*, *Hif1a*, and *Chuk* transcript levels were assessed by qRT-PCR. **b** Naïve CD4^+^ T cells were isolated from WT and *Pparα* KO mice and cultured under Th17 polarizing conditions for 72 h. IKKα expression in naïve CD4^+^ T cells (left) or Th17 cells (right) from WT and *Pparα* KO mice was assessed by western blot. **c** Naïve CD4^+^ T cells from WT and *Ppara* KO mice were polarized under Th17 condition for 72 h and immunofluorescence staining was performed as described in the method section, and confocal imaging was acquired. IKKα alone (left); nucleus (middle), and merged view (right). **d** Naïve CD4^+^ T cells from WT and *Pparα* KO mice were polarized under Th17 condition for 72 h, and chromatin immunoprecipitation (ChIP) assay was performed by utilizing isotype-match IgG (grey bars), anti-IKKα (red bars), anti-RORγt (blue bars), and anti-phospho-histone H3 (yellow bars) antibodies. qRT-PCR was performed utilizing primers flanking putative RORγt binding region or non-binding region as described in the method section. **e** 293 T cells were transfected with Myc-tagged RORγt expression plasmids (Myc-RORγt), HA-tagged IKKα expression plasmids (HA-IKKα), or both. Co-immunoprecipitation assay was performed by utilizing anti-Myc antibody (IP: Myc) on cell lysates, and the western-blot of either HA or Myc is shown (IB). The input amount is also shown in the bottom. Results are shown as the means ± SEM of five independent trials except for picture b, where results are shown as the means ± SEM of three independent trials. Unpaired student t-tests were used for statistical analysis representing *P < 0.05, and **P < 0.01.

To investigate the molecular basis of *Pparα*-dependent suppression of Th17 development, we analyzed known regulators of Th17 development. It is known that phosphorylation of signal transducer and activator of transcription (STAT3), downstream of IL-6 signaling, is critical for Th17 development^15,37^. *Pparα* KO CD4^+^ T cells showed comparable STAT3 phosphorylation  and *Il17a* levels compared to WT CD4 + T cells (Supplementary Fig. 2a, b). This lack of effect on STAT3 activation contrasts with the results of *Pparα* KO^38^. Expression of RORγt (gene name: *Rorc)* and RORα (gene name: *Rora*), master regulators of the Th17 lineage^2,39^, was also assessed in CD4^+^ T cells from WT and *Pparα* KO mice. Interestingly, despite the elevated IL-17 production seen in KO-derived T cells, we observed a slight increase in the expression of RORγt, while the expression of RORc was not affected in the CD4^+^ cells isolated from the *Pparα* KO mice (Fig. 2a). Also, in line with our previous findings^15^, hypoxia-inducible factor-1 (HIF-1α, gene name: *Hif1α*) expression was higher in the enhanced Th17 population lacking *Ppara* (Fig. 2a). Since HIF-1 levels can be stabilized, even under normoxic conditions, by TCR signaling-dependent transcriptional activation and NFKB activity, it is possible that the enhanced *Hif1α* level we found could be the result of enhanced NFKB signaling in the absence of PPARα, and that the enhanced IL-17 could be due to *Hif1α* elevation. Supporting this, we found an elevated NFKB activity in the absence of *Pparα*, which did not result from elevated *Hif1α* levels. We found increased expression of *Chuk*, conserved helix-loop-helix ubiquitous kinase, which is also known as an inhibitor of NFKB kinase subunit alpha (IKKα), after the loss of *Pparα* (Fig. 2a, b).

Considering the role of IKKα in mediating the *Il17a* phenotype^40^, we determined the protein expression of IKKα in the naïve CD4^+^ T cells and Th17 cells. We found that *Pparα*^*-/-*^ substantially increased the protein expression of IKKα in both naïve T and Th17 cells (Fig. 2b; Supplementary Fig. 5). Furthermore, given that IKKα can translocate into the nucleus^41^, we tested whether enhanced IKKα expression levels in *Pparα* KO Th17 cells correlate with its nuclear localization. Naïve CD4^+^ T cells were isolated from WT and *Pparα* KO mice and cultured under Th17 polarizing conditions for 72 h. Immunofluorescence assay indicates a strong perinuclear localization of IKKα in the WT Th17 cells (Fig. 2c, top). In contrast, IKKα expression was uniformly distributed throughout the center of the nucleus in *Pparα* KO Th17 cells (Fig. 2c, bottom), suggesting its physical proximity to active chromatins. Furthermore, the chromatin immunoprecipitation (ChIP) assay determined that RORγt binding to its well-known *Il17a* regulatory region was comparable in both WT and *Pparα* KO Th17 cells (Fig. 2d, blue bars). Still, IKKα binding to the same regulatory region was enhanced in Th17 cells from *Pparα* KO mice (Fig. 2d, red bars). Meanwhile, enhanced phosphorylation of histone H3 H3Ser10 was associated with increased IKKα binding in Th17 cells from *Pparα* KO compared to WT mice (Fig. 2d, yellow bar). To further strengthen the epigenetic modification of RORγt in Th17 cells from *Pparα* KO mice, we carried assay to determine H3K27ac enrichment at the RORγt. The results indicated that H3K27ac enrichment was significantly increased at RORγt binding region in Th17 cells from *Pparα* KO mice (Supplementary Fig. 3a). This supported our findings suggesting the enhanced nuclear localization of IKKα in *Pparα*^*-/-*^. During *Il17a* transcriptional regulation, RORγt can be associated with various transcriptional complexes, including *Hif1α* and RORα^15,39^. Given that *Il17a* doesn’t contain strong PPREs, we further analyzed whether IKKα can be associated with RORγt (Fig. 2e; Supplementary Fig. 5). Co-immunoprecipitation data indicated that there is a strong binding between IKKα and RORγt (Fig. 2e, lane 4; Supplementary Fig. 6). Overall, these data demonstrated that IKKα binds to the promotor region of RORγt to form a transcriptional complex that activates the transcription of IL-17.

**Fig. 3:**
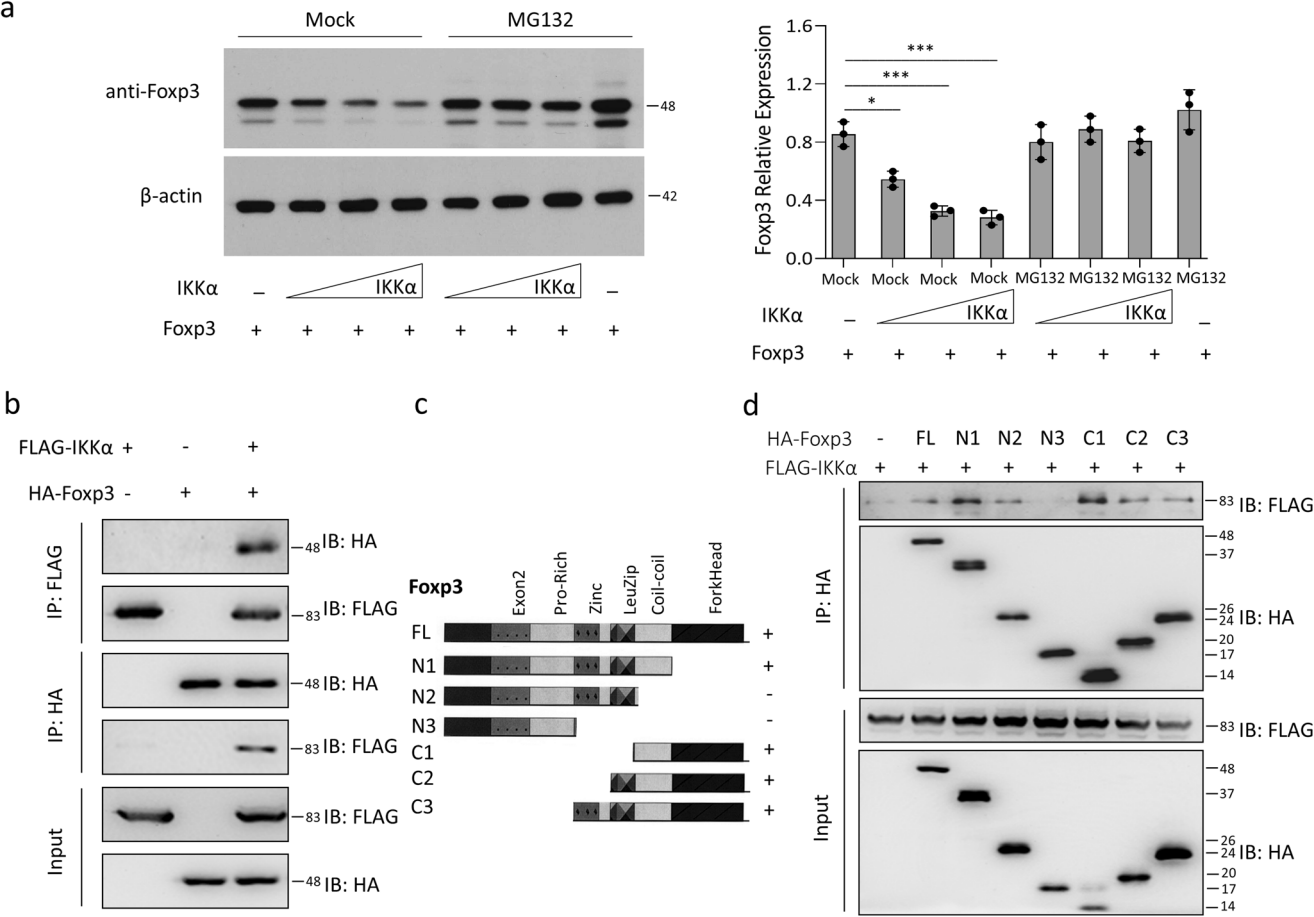
IKKα binds to the Foxp3 and leads to its proteasomal degradation. **a** 293 T cells were transfected with Foxp3 expression plasmids together with increasing concentrations of IKKα overexpression plasmid in the presence of vehicle or MG132 (protease inhibitor). After 48 h of incubation, the cells were lysed and immunoblotted for Foxp3 (top) and β-actin (bottom). **b** 293 T cells were transfected with HA-tagged Foxp3 expression plasmids (HA-Foxp3), Flag-tagged IKKα expression plasmids (Flag-IKKα), or both. Co-immunoprecipitation assay was performed utilizing anti-Flag antibody (IP: Flag) or anti-HA antibody (IP: HA) on cell lysates and western-blot of either HA (Foxp3) or Flag (IKK) is shown (IB). The input amount is also shown in the bottom. **c** Full-length and truncated protein domains of Foxp3 are shown in schematic diagrams. N1, N2 and N3 represent Foxp3 constructs missing C-terminus domains, while C1, C2 and C3 represent Foxp3 constructs missing N-terminus domains. **d** Truncated Foxp3 constructs tagged with HA were transfected into 293 T cells together with Flag-IKKα and co-IP was performed with anti-HA-antibody (IP: HA). Subsequent western blot analysis of either HA (Foxp3) or Flag (IKKα) is shown. Results are shown as the means ± SEM of three independent trials. Unpaired student t-tests were used for statistical analysis representing *P < 0.05, and ***P < 0.001.

Meanwhile, considering the role of IKKα in regulating Foxp3, we speculated the potential interaction of Foxp3 with IKKα. To validate this, we first used increasing concentration of IKKα plasmid in the presence of MG132 (protease inhibitor). The results indicated that an increase in the concentration of IKKα plasmid leads to a decrease in the expression of Foxp3 (Fig. 3a; Supplementary Fig. 7). Meanwhile, co-immunoprecipitation assay indicated the interaction between Foxp3 and IKKα (Fig. 3b; Supplementary Fig. 8). Furthermore, to identify the domain of Foxp3 interacting with IKKα, we generated several truncations containing the functional domains of the Foxp3 (Fig. 3c), and performed co-immunoprecipitation. The results indicated that FLAG-IKKα binds with the N1 and C1 region of HA-Foxp3 (Fig. 3d; Supplementary Fig. 9). To further strengthen the relationship between IKKα and Foxp3 in native conditions, we conducted endogenous Co-IP using the plasmids overexpressing IKKα and Foxp3. The data indicated that IKKα and Foxp3 also interacted endogenously (Supplementary Fig. 3b) Thus, we established that an increase in IKKα can lead to a decrease in Foxp3 expression through post-translational modification in term of proteasomal degradation of Foxp3 by phosphorylation.

### Loss of *Pparα* aggravates the EAE injury by altering the Th17/Treg balance

**Fig. 4:**
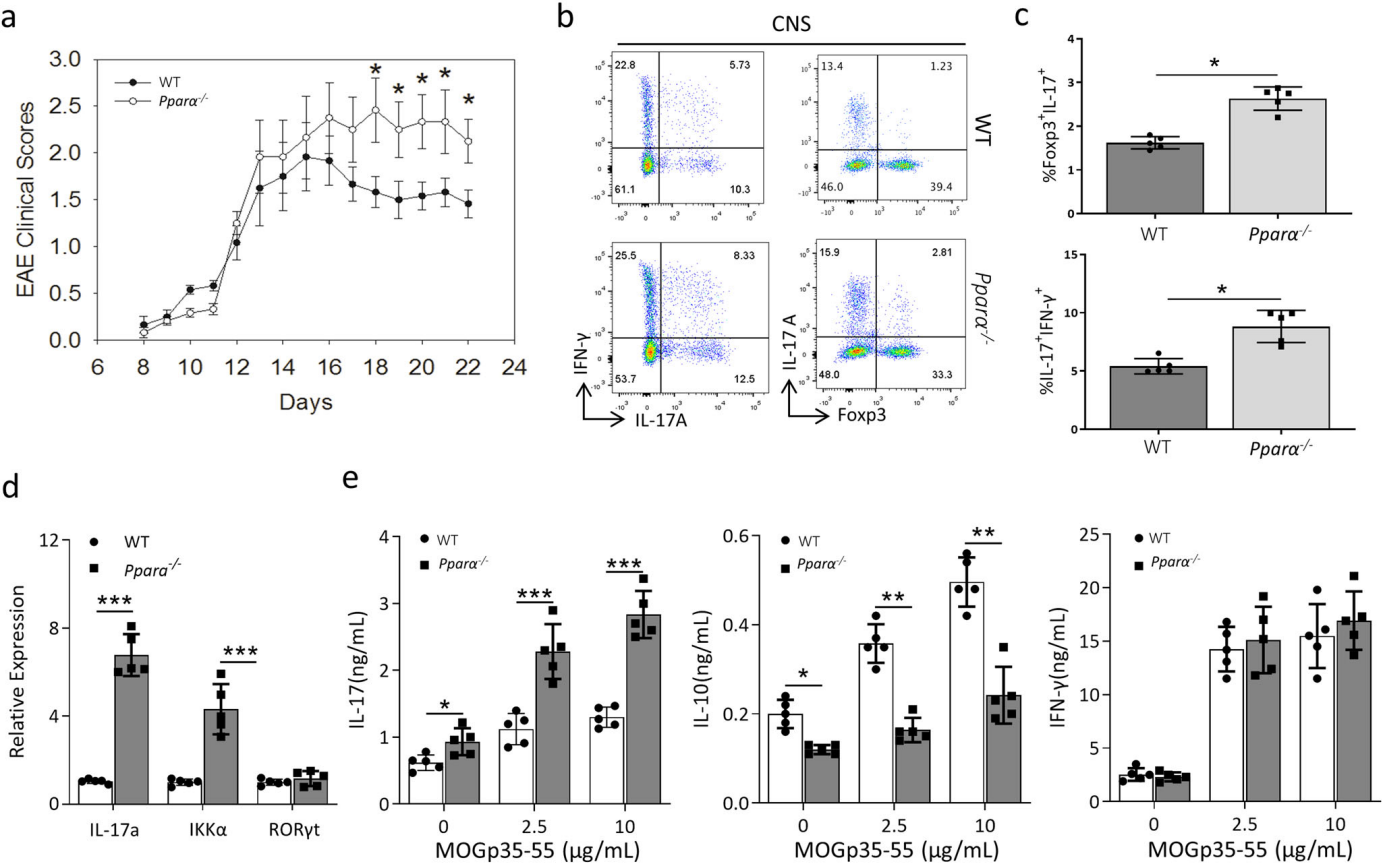
Pparα mediates the progression of EAE by modulating the population of Th17 cells. **a** Clinical scores of WT and *Pparα* KO mice immunized with 100 ug MOG35-55 peptide (MOGp35–55) in complete Freund’s adjuvant. Clinical scores are shown as the means ± SEM of *n* = 6 mice per group. Unpaired student t-test were used for statistical analysis representing *P < 0.05. **b** Representative flow-plots of IFN-γ, IL-17A and Foxp3 expression in lymphocytes from the brain of WT and *Pparα* KO mice. **c** Foxp3^+^ IL17A^+^ (top) or IFN-γ^+^ IL-17A^+^ (bottom) double positive CD4^+^ T cells. **d** qRT-PCR analysis analyzed the expression of IL-17A, IKKα and RORγt from the mononuclear cells isolated from the brain of WT and *Pparα* KO mice on day 22 following the induction of EAE. **e** ELISA was performed to analyze the levels of IL-17A, IL-10 and IFN-γ in the splenocytes isolated from WT and *Pparα* KO mice at 22 days-induced EAE model after stimulation with MOGp35–55 or medium alone. In-vivo data are shown as the means ± SEM of *n* = 5 mice per group. For in-vitro experiments, results are shown as the means ± SEM of five independent trials. Unpaired student t-test were used for statistical analysis representing *P < 0.05, **P < 0.01, and ***P < 0.001.

Finally, we utilized an EAE model and investigated whether *Pparα* deficiency can alter Th17 and Treg developmental balance in vivo. WT and *Pparα* KO mice were immunized with MOG peptide, and clinical paralysis was monitored and assessed for 22 days (Fig. 4a). Beginning from day 14, *Pparα* KO mice developed more severe paralysis, which was maintained rest of the experiment (Fig. 4a). On day 22, we isolated CD4^+^ T cells infiltrating the brain (CNS), and evaluated the expression of IL-17A, Foxp3 and IFN-γ expression by using flow cytometry (Fig. 4b). In association with more severe paralysis^42^, *Pparα* KO mice have significantly increased percentage of IL-17A^+^ IFN-γ^+^ double positive (DP) CD4^+^ T cells in the CNS compared to WT mice (Fig. 4c). At the same time, we observed a small increase in IL-17A single positive (SP) subset with a reduction in Foxp3 SP subset among CD4^+^ T cells in *Pparα* KO mice. The percentage of IL-17A^+^ Foxp3^+^ DP CD4^+^ T cells was significantly higher in *Pparα* KO mice (Fig. 4c), demonstrating enhanced IL-17A expression in the absence of *Pparα*^43^. In contrast, such difference was not seen in the Treg/Tconv ratio of CD4^+^ T cells from the spleen after stimulation (Supplementary Fig. 2c). To further analyze the expression of IL-17a, IKKα, and RORγt in the EAE-induced animal model, mononuclear cells were isolated from the brain of WT and *Pparα* KO mice on day 22 following induction of EAE, and qRT-PCR was performed. We found that the levels of IL-17A and IKKα were significantly upregulated. In contrast, the level of RORγt was not changed in *Pparα* KO mice as compared to WT mice (Fig. 4d). Meanwhile, the cytokine production of splenocytes isolated from WT and *Pparα* KO mice at day 22 after EAE induction was analyzed after stimulation with MOG35–55 peptide (MOGp35–55) or medium alone using the ELISA. The results indicated that the level of IL-17A was significantly increased in the splenocytes isolated from *Pparα* KO mice at 0, 2.5, and 10 μg/mL doses of MOGp35–55 compared to WT (Fig. 4e). However, the level of IL-10 was significantly decreased in cells isolated form *Pparα* KO mice at 0, 2.5 and 10 μg/mL doses of MOGp35–55 (Fig. 4e). Interestingly, we didn’t observe any change in the level of IFN-γ between *Pparα* KO and WT group (Fig. 4e). This was consistent with the finding that brain-resident MOG-specific T cells are associated with the development of EAE^44^.

## Discussion

Many recent studies have recognized the role of nuclear receptors during Th17 and Treg development. Nuclear receptors, including PPARs, utilize unique modes of gene regulation called trans-repression and trans-activation^45^, which are different from other transcription factors. Rather than acting as a direct transcriptional activator or suppressor, PPARs regulate gene expression by recruiting epigenetic modifiers or interfering with other signaling pathways^46^. PPARα appears to follow this mechanism during Th17 development, recruiting co-repressors, silencing mediators for retinoid and thyroid receptors (SMRT), and nuclear receptor co-repressor (NcoR)^25,47^. Here, it has been illustrated that genetic ablation of *Pparα* can result in enhanced Th17 responses and exacerbated EAE. Meanwhile, we have found a modest increase in IL-17A expression in CD4^+^ T cells under Th17 polarizing conditions in *Pparα* KO mice.

Similar to *Pparγ*, other studies have found that *Pparα* can regulate Th17 responses gender-dependently^29^. Furthermore, gemfibrozil, a *Pparα* agonist, can enhance Th2 responses and thus ameliorates EAE symptoms^48^. Nevertheless, the mechanism of action of *Pparα* is not fully established, and to the best of our knowledge, our data present a novel molecular mechanism of *Pparα* regulating Th17. NF-κb signaling has been known to play roles in Th17 and Treg development^49–51^, yet its molecular mechanism has not been fully established. IKKα is a serine/threonine protein kinase member that mediates the classical and alternative NF-κb signaling pathway^52^. In the NF-κb dependent pathway, IKKα-mediated phosphorylation can signal proteasomal-mediated degradation of IκB or p100. Independent of the NF-κb pathway, IKKα also contains a nuclear localization signal sequence that allows its translocation and gene regulation by phosphorylation of histone H3^41,53^. It has been known that *Pparα* can inhibit NF-κB-activation by suppressing IKKα activity^54^, and our finding provides mechanistic details of *Pparα* interaction with the NF-κB pathway. We found that *Ppara* KO naïve CD4^+^ T cells have elevated basal levels of IKKα expression compared to WT CD4^+^ T cells. Such difference was maintained after naïve T cells differentiated under Th17 polarizing conditions. Yet, it is not clear how *Pparα* inhibits IKKα expression levels in T cells, but a similar mechanism that regulates IκB expression may play a role^55^.

PPARs can directly bind to peroxisome proliferator response elements (PPREs) in proximal regulatory regions and regulate gene expression^56^. However, *Il17a* regulatory regions do not contain PPREs as previously reported^25^, leaving the mechanism of *Pparα*-dependent regulation of Th17 differentiation both unknown and potentially complex. Numerous studies have reported that IKKα-dependent phosphorylation of histone H3 is critical for IL-17A expression in CD4^+^ T cells^40^. We provide evidence that *Pparα* is an upstream regulator of IKKα expression and nuclear translocation during Th17 development. Furthermore, our data suggest that altered nuclear distribution of IKKα in *Pparα* KO Th17 cells in association with RORγt allows its transactivation of *Il17a* expression. Thus, it is plausible that *Pparα* may physically interact and sequester IKKα to the perinuclear region, inhibiting the interaction between IKKα and RORγt. Because RORγt is highly expressed in the Th17 subset, this proposed mechanism may also explain the specific effects of *Pparα* on Th17 differentiation.

The balance of Th17 and Treg is important for autoimmunity and immune tolerance^15^. However, the role of IKKα and *Pparα* in balancing Th17/Treg has not been understood. A previous finding indicates that IKKα deficiency in CD4 cells decreased their response to TCR stimulation and reduced the number of Tregs in peripheral lymph nodes and thymus^57^. Meanwhile, IKKα deficient Tregs showed failure in their ability to lower the expression of IFN-γ and elevation of IL-17 in naïve CD4 cells^57,58^. However, in our study, we observed an increased IKKα expression in the naïve CD4^+^ cells from *Pparα* KO mice cultured under Th17 polarization suggesting the role of IKKα in Th17 polarization. Meanwhile, we also found that forced expression of IKKα significantly lowers the expression of Foxp3, thus influencing the differentiation and function of Treg. Therefore, in accordance with previous findings^40,57^, our study urges that IKKα plays a critical role in the Th17 development and Th17/Treg balance by enhancing the transcription of IL-17 and proteasomal degradation of Foxp3. Interestingly, TGF-β regulates the differentiation of both Th17 and Tregs. In naïve T cells, TGF-β elevates the Tregs development, and inhibits Th17 development. However, TGF-β fails to induce Tregs under IL-6 conditions; instead, it promotes Th17 development by enhancing RORγt expression^59^. Thus, our study suggests that *Pparα* KO or inhibition of Foxp3 could enhance Th17 development. Meanwhile, further studies are required to investigate how inhibiting the expression of *Pparα* in immune cells can influence disease activity.

Besides the direct role of IKKα in the post-translational modification of Foxp3, as shown by proteasomal degradation of Foxp3 by IKKα in our study, IKKα also regulates the expression and function of Foxp3 in an NF-κB-dependent manner. However, in NF-κB-dependent regulation of Foxp3, c-Rel, a member of NF-κB, is particularly important for regulating the expression of Foxp3 at the transcriptional level^60,61^. Meanwhile, NF-κB and c-Rel also play an indispensable role in the development of Th17 cells^50,62^. However, we found that independent of NF-κB, IKKα can lead to the proteasomal degradation of Foxp3 and the transcription of IL-17. It was due to the nuclear translocation of IKKα in our* Pparα* KO mice. It has been previously observed that the nuclear translation of IKKα can also directly influence cell functions^63^. However, our study found that the absence of *Pparα* upregulates the IKKα, leading to post-translational changes in the Foxp3 through phosphorylation. Meanwhile, it needs further investigation to understand the cell-specific effect of *Pparα* in Treg and Th17 development.

Of note, we observed an increase in the Foxp3^+^ IL17^+^ and IL-17^+^ IFN-γ^+^ DP CD4 cells in the EAE model induced in *Pparα* KO mice. These DP T cells mediate the differentiation of Tregs and Th17 cells in the presence of cytokine milieu, i.e., IL-6 and TGF-β^64,65^. Increased DP cells act as a transient population and enhance the proinflammatory condition and disease severity during the pathogenesis of EAE in *Pparα* KO mice. A previous study also indicated that Foxp3 directly binds to the RORγt and regulates the activity and transcription of RORγt and IL-17 mRNA, respectively^66^. Conversely, an increase in double-positive cells further strengthens our hypothesis that IKKα, in the absence of Pparα, aggravates the progression of EAE by supporting the differentiation of Th17 through the proteasomal degradation of Foxp3.

Adoptive transfer of Tregs is a potent immunotherapy against various diseases^67,68^. It has been elucidated that the adoptive transfer of Tregs somehow prevents or decrease the severity of experimental autoimmune encephalomyelitis (EAE) and neurological symptoms^69^. Whereas the depletion of Tregs can aggravate the EAE^70^. However, we observed a little decrease in the population of Foxp3^+^ Tregs in *Pparα* KO mice induced with EAE. This was accompanied by the increase in Foxp3^+^ IL17^+^ and IL-17^+^ IFN-γ^+^ DP CD4 cells which alleviated the disease in KO mice.

Molecular regulators that regulate Th17 and Treg development have been identified^71^. TGF-β1 driven Foxp3 can inhibit RORγt activity in a dose-dependent manner and suppress Th17 development^59,66^, and its expression levels can affect Th17 development. HIF-1α can post-translationally modify Foxp3 expression during Th17 development while enhancing IL-17A expression^15^. Also, acetyl-CoA carboxylase 1 (ACC1) is required for fatty acid synthesis and optimal Th17 development^72^. IKKα’s interaction with RORγt presents another mechanism underlying the Th17/Treg developmental balance. Our results indicate that *Pparα* and IKKα are therapeutic targets for inhibiting the Th17 subset in major autoimmune diseases^73^. Overall, our study provides an interesting insights into *Pparα* and IKKα-dependent degradation of Foxp3 and transcriptional enhancement of *Il17a* (Supplementary Fig. 1d). It is suggested that during the activation of TCR signaling in naïve CD4^+^ T cell, there is an increase in the activity of *Pparα*. This activation of *Pparα* inhibits the expression and functions of IKKα. IKKα is involved in the proteasomal degradation of Foxp3 and the upregulation of *Il17a* by binding Foxp3 and RORγt, respectively. Thus, to the best of our knowledge, our results suggest a novel therapeutic approach to treating autoimmune and inflammatory diseases.

## Methods

### Mouse strains

*Ppara* KO mice on C57/BL6 background were purchased from the Jackson Laboratory. *Stat3* f/f; *Cd4*-cre mice were previously described. *Ppara* KO mice were crossed to *Stat3* f/f; CD4-cre to generate DKO mice. 6–8 weeks-old male mice were used for experiments. All animal experiments complied with and were approved by the Johns Hopkins and SIAT Animal Care and Use Policy.

### In vitro T-cell differentiation

Naïve CD4^+^ T cells (CD4^+^ CD25^-^ CD62L^Hi^) were sorted on a FACS Aria high speed sorter. The sorted cells were activated with plate-bound α-CD3 (10 ug/mL) and soluble α-CD28 (2 ug/mL) with the following polarizing conditions: Th1 (IL-12 (10 ng/mL), IL-4 (10 ug/mL)), Th2 (IL-4 (10 ng/mL), IFN-γ (10 ug/mL), IL-12 (10 ug/mL)), Th17 (IL-6 (10 ng/mL), TGF-β (1.25 ng/mL), IL-23 (10 ng/mL), IL-1β (10 ng/mL), IFN-γ (10ug/mL), IL-4 (10 ug/mL)), Treg (TGF-β (5 ng/mL), IL-2 (100 IU/mL)).

### Flow cytometry

For extracellular staining, harvested cells were washed and incubated in PBS containing 1% FBS containing the below fluorochrome-conjugated antibodies in a U-bottom 96-well plate. For intracellular cytokine staining, harvested cells were re-challenged in PMA and Ionomycin in the presence of Golgi-Plug (BD Biosciences). After 5 hours of incubation, the cells were fixed/permeabilized (eBioscience) and incubated with antibodies. The following antibodies were used for the flow cytometric assay: IFN-γ PE, IFN-γ APC, IL-13 PE, IL-17 APC (BD Bioscience), IL-2 APC (BD Pharmingen), Foxp3 PE (eBioscience). The gating strategy used has been described in Supplementary Fig. 4. For cellular proliferation, Cell Trace CFSE cell proliferation kit (Invitrogen) was used per the manufacture’s manual.

### Co-immunoprecipitation and Immunoblotting

293 T cells were transfected with the plasmids overexpressing IKKα, RORγt, Foxp3, and various fragments of Foxp3 using Lipofectamine 2000 (Invitrogen, Waltham, MA, USA). Plasmids were co-transfected or transfected individually depending on the requirement for endogenous or exogenous Co-IP experiment. After 48 h of transfection, total protein was extracted using the RIPA lysis buffer. Proteins mixtures were then incubated with following antibodies: anti-HA (Sigma-Aldrich, 1:2000), anti-Flag (Sigma-Aldrich, 1:5000), or anti-Myc (Sigma-Aldrich, 1:2000) to bind with the protein complexes. Later, Dynabeads® Protein G (Invitrogen, Cat.No.100.04D) were utilized to pulldown the protein complexes. Finally, the samples were centrifuged to remove the supernatant, washed with IP buffer three times, eluted in SDS-loading buffer, and heated at 100 °C for 8 min.

Samples were then subjected to western blotting for separation using 10% SDS-PAGE. After the electrophoresis, proteins were transferred to a PVDF membrane which was ultimately blocked with 5% skim milk for 2 h. After blocking, membrane was incubated with respective primary antibodies for overnight. Next day, membrane was washed and incubated with HRP-conjugated secondary antibodies. After washing, membrane was exposed using ECL system. Protein sizes in blot images were calculated by resolving the pre-stained potein marker using electrophoresis. ImageJ was used to calculate the intensity of protein bands.

### Small molecule inhibitors

Fenofibrate and Gemfibrozil were purchased from Sigma-Aldrich and dissolved in DMSO. Dilutions were made in DMSO before being added to cultures to make indicated final concentrations in culture.

### EAE induction

Age- and sex-matched mice were immunized with 100 ug MOG35-55 peptide (MOGp35-55) in complete Freund’s adjuvant (Sigma-Aldrich) in the rear flank *s.c*. The mice were further challenged with 250 ng of pertussis toxin (List Biological) on day 0 and day 2. Paralysis in mice were monitored and scored as previously described criteria.

### Quantitative real-time PCR

RNA was extracted using Trizol (Invitrogen) followed by cDNA synthesis reaction using SuperScript III (Invitrogen) in a 20 ul reaction/well. The same amount of RNA was used in each cDNA synthesis reaction measured by NanoDrop Spectrophotometer (ThermoScientific). The same volume of cDNA per sample was prepared for qRT-PCR analysis using SYBR Green (Pierce) and the indicated primers to assess transcript levels of each gene. The primer sequences were included in the Supplementary Table 1.

### ELISA

The concentration of IL-17, IL-10, and IFN-γ from the splenocytes isolated from WT and *Pparα* KO mice at 22 days after EAE induction and stimulated with MOGp35–55 or medium alone. Samples were detected for respective cytokines using the ELISA kit. Values were detected using a micro-plate reader at OD450.

### CFSE dilution assay

CD4^+^ T cells were isolated, as mentioned earlier. Cells were labeled with 1 mM CFSE (Molecular Probes) in PBS and incubated at 37 °C for 3 min. Ultimately cells were washed and seeded in a 96-well plate, which was previously coated with 10 mg/mL anti- IgG Fc-specific Ab along with 1 mg/mL anti-CD3 and 0.25 mg/mL anti-CD28 at 4  °C overnight. Cells were then harvested and analyzed for IL-2 and IFN-γ using flow cytometry.

### Chromatin immunoprecipitation assay

Relative histone modification levels in Th17 cells from WT and* Paprα* KO mice were detected using in-vitro ChIP assay. Cells were cross-linked using disuccinimidyl glutarate (DSG) for 30 min followed by 1% methanol-free ultrapure formaldehyde for 10 min at RT. Glycine was then added for 5 min at 125 mM final concentration. After fixation, chromatin samples were sonicated with a Diagenode Bioraptor to generate small fragments of 200–1000 bp, with a peak of intensity of about 500 bp. The supernatant after centrifugation was diluted by ChIP dilution buffer and precleared with protein A–agarose beads, which was then immunoprecipitated. Chromatin was immunoprecipitated with antibodies against IgG, anti-IKKα, anti-RORγt, anti-p-H3K27ac, and anti-p-H3Ser10. The immunoprecipitated DNA was isolated by Universal DNA purification kit (TIANGEN, DP214-03). Purified DNA was analyzed by qRT-PCR with specific primers.

### Statistics and reproducibility

In-vivo data are shown as the means ± SEM of *n* = 6 mice per group. For in-vitro experiments, results are shown as the means ± SEM of three or more independent trials as represented in figure legends. The raw data related to this study has been presented in Supplementary Data 1. Data were analyzed and compared through the GraphPad Prism 8.0 by utilizing an unpaired Student’s t-test, and for the significance, *p*-value of <0.05 was acquired.

### Reporting summary

Further information on research design is available in the Nature Portfolio Reporting Summary linked to this article.

## Supplementary information

Supplementary Information

Description of Additional Supplementary Files

Supplementary Data 1

Reporting Summary

## Data Availability

The Supplementary Figs. 5–9 are the source uncropped Western blot images with size marker shown in this manuscript. The Supplementary Data 1 contain all the statistical source data for the graphs presented in the manuscript. All other data is available from the corresponding author upon reasonable request.
