# Supplementary material for: Pparα knockout in mice increases the Th17 development by facilitating the IKKα/RORγt and IKKα/Foxp3 complexes
Source: Commun Biol. 2023 Jul 14;6:721. doi: 10.1038/s42003-023-05104-6 (PMC10349144; doi:10.1038/s42003-023-05104-6)
Supplement: Supplementary file 1 — Supplementary Information [file 42003_2023_5104_MOESM1_ESM.pdf]

Pparα knockout in mice increases the Th17 development by facilitating the IKKα/RORγt and IKKα/Foxp3 complexes

Ping Wei[1], Wei Kou[1,2], Juan Fu[2], Zuojia Chen[3], and Fan Pan[3,4*]

[1.] Department of Otolaryngology, Ministry of Education Key Laboratory of Child Development and Disorders, National Clinical Research Center for Child Health and Disorders (Chongqing), China International Science and Technology Cooperation base of Child Development and Critical Disorders, Children's Hospital of Chongqing Medical University, Chongqing, China

[2.] Department of Oncology, Sidney Kimmel Comprehensive Cancer Center, Johns Hopkins University School of Medicine, Baltimore, MD, USA

[3.] Experimental Immunology Branch, National Cancer Institute, NIH, Bethesda, MD, USA

[4.] Shenzhen Institute of Advanced Technology (SIAT), Chinese Academy of Sciences (CAS), 1068 Xueyuan Avenue, Shenzhen 518055, P. R. China

*Corresponding author:

Fan Pan (E-mail: fan.pan@siat.ac.cn; ORCiD: 0009-0008-6325-0089)

Running Title: Pparα-dampens Th17 development by controlling IKKα

Supplementary Figure 1

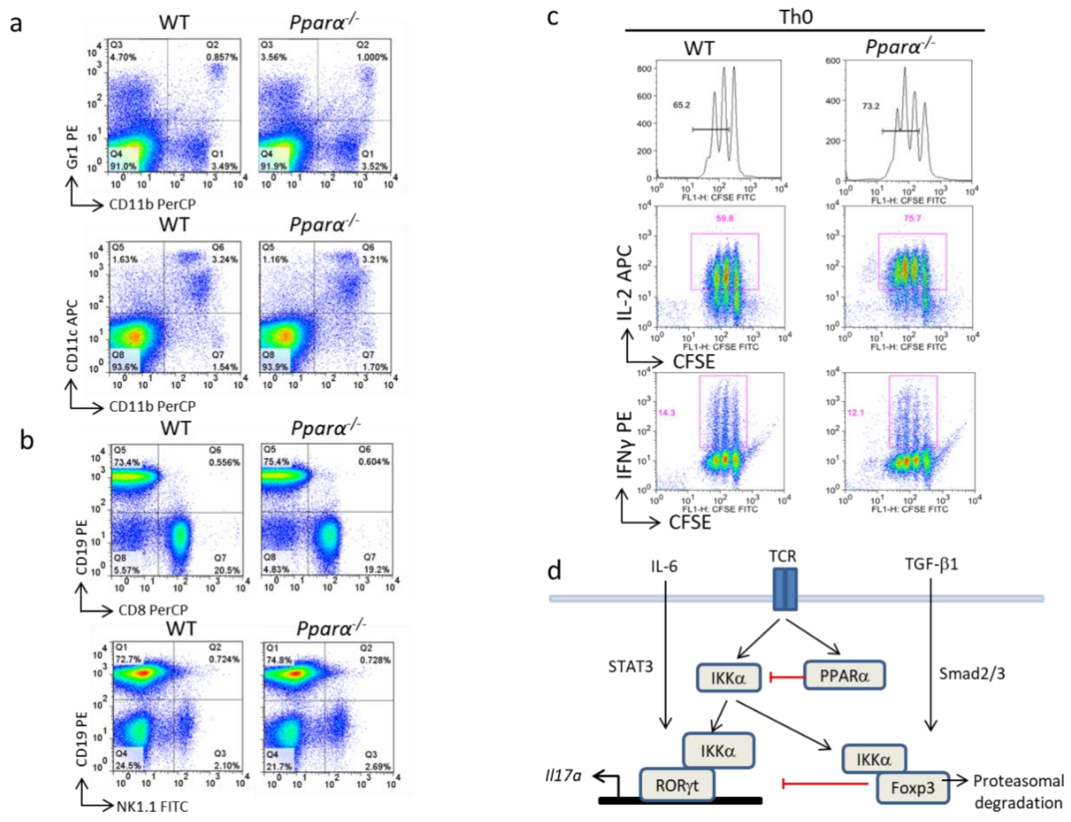

Supplementary Figure 1. Effect of CD4+ specific knock-out of PPARα on the profile of immune cells. (a) Representative flow-plots of Gr1+ and Cd11c+ cells in the spleen and thymus of *Pparα* KO mice. (b) Representative flow-plots of CD8+, CD19+ and NK1.1+ cells in the spleen and thymus of PPARα KO mice. (c) CFSE dilution representing the proliferation of na ïve CD4+ T cells and the expression profile of IL-2 and IFNγ in the naïve CD4+ T cells from WT and *Pparα* KO mice cultured under non-polarizing (Th0) conditions. (d) Schematic illustrations of PPARα and IKKα mediated regulation of IL-17 and Foxp3 expression during Th17 development. In-vivo data are shown as the means ± SEM of n=6 mice per group. For in-vitro experiments, results are shown as the means ± SEM of five independent trials.

Supplementary Figure 2

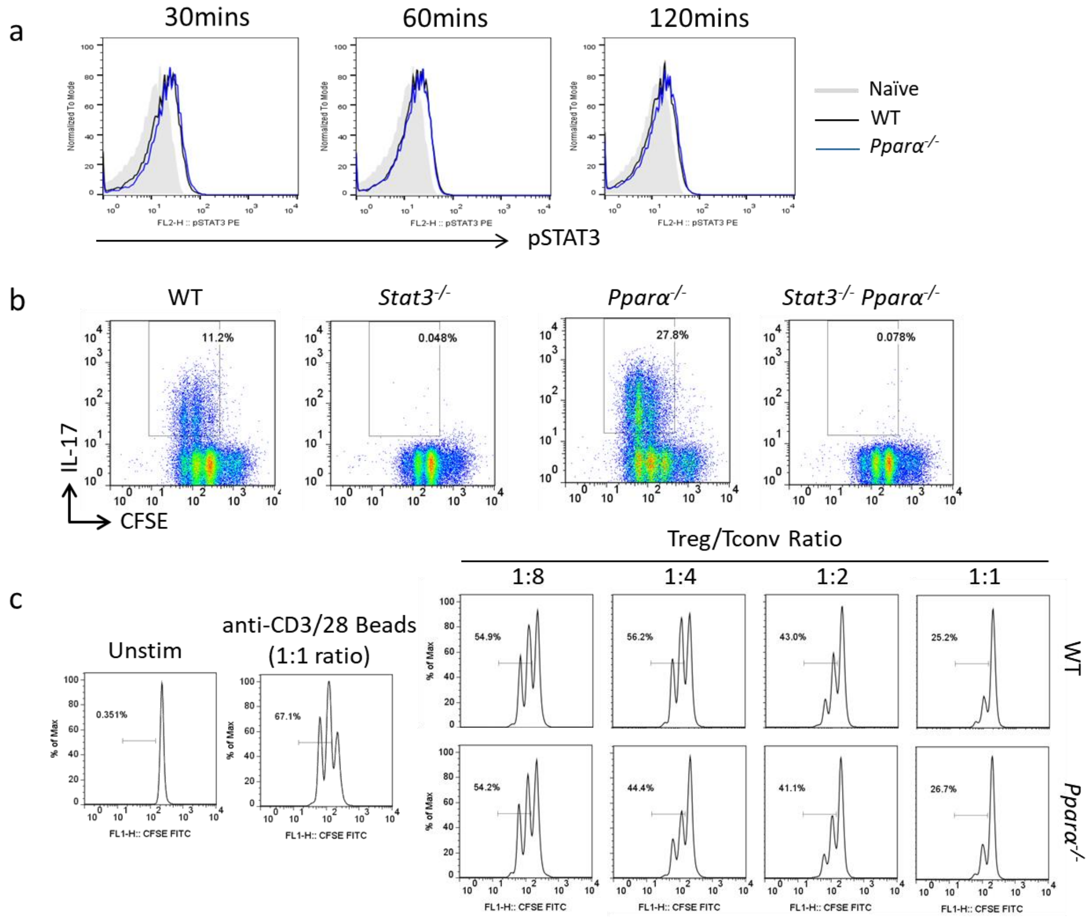

Supplementary Figure 2. Influence of *Ppara* KO on the phosphorylation of STAT3 and Treg ratio. (a) Expression of pSTAT3 in CD4+ cells isolated from PPARα KO mice. (b) CFSE dilution representing the expression profile of IL-17 in the naïve CD4+ T cells from WT and *Ppara* KO mice cultured under non-polarizing (Th0) conditions. (c) Ratio of Treg and Tconv in T cells isolated from PPARα KO mice after stimulation with anti-CD3 and anti-CD28 antibodies. For in-vitro experiments, results are shown as the means ± SEM of five independent trials.

Supplementary Figure 3

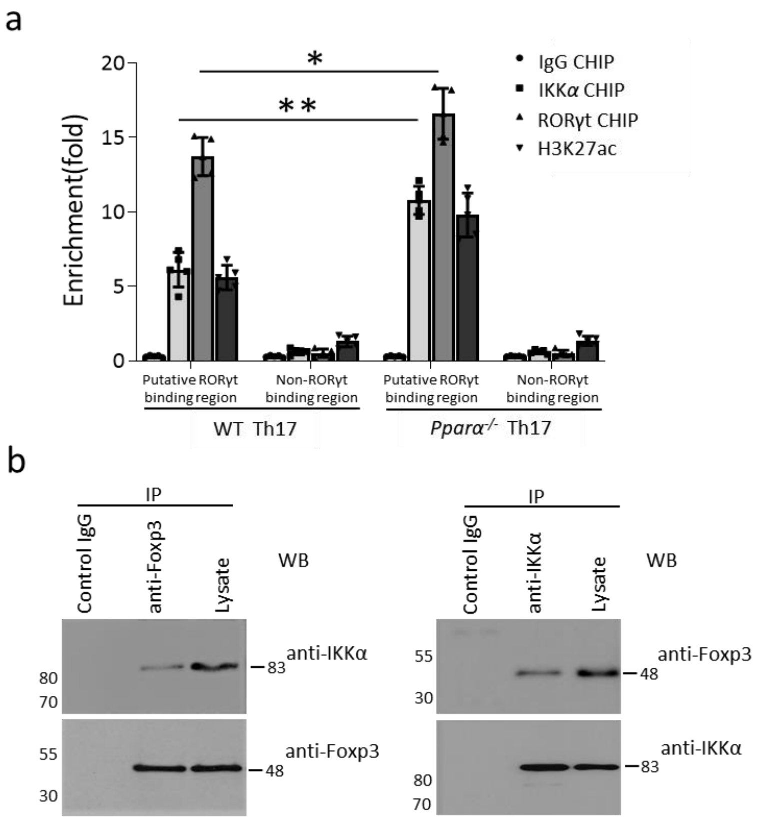

Supplementary Figure 3. *Ppara* modifies the RORγt and Foxp3. a) *Ppara* KO increases the H3K27ac enrichment at RORγt binding region in Th17 cells. Na ïve CD4+ T cells were isolated from WT and *Ppara* KO mice, and polarized under Th17 condition for 72hrs. Chromatin immunoprecipitation (ChIP) assay was conducted utilizing isotype-match IgG (grey bars), anti-IKKα (red bars), anti-RORγt (blue bars), and anti-pH3K27ac (yellow bars) antibodies. qRT-PCR was performed utilizing primers flanking putative RORγt binding region or non-binding region as described in the method section. Results are shown as the means ± SEM of duplicate experiments and representative of three independent trials. Unpaired student t-tests were used for statistical analysis representing * $P < 0.05$, and ** $P < 0.01$. b) Endogenous interaction of Foxp3 and IKKα. Co-immunoprecipitation assay was performed using the anti-Foxp3 to pull down IKKα (Left panel), and anti-IKKα to pull down Foxp3 (Right panel) endogenously. Results are shown as five experimental replicates.

Supplementary Figure 4

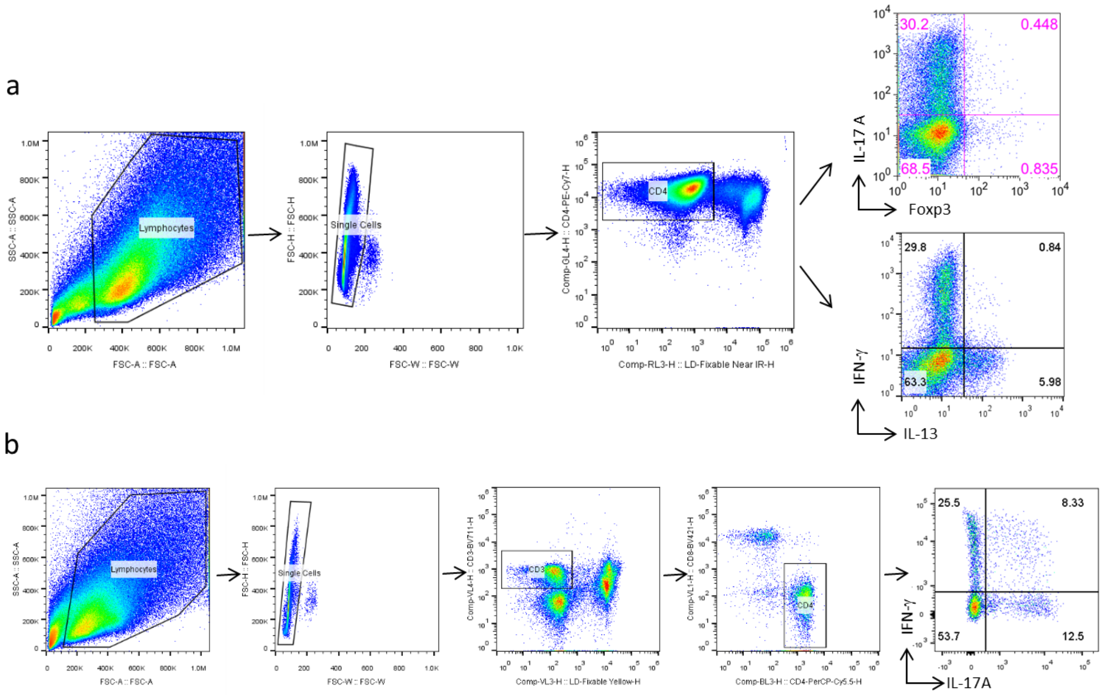

Supplementary Figure 4: Gating strategy for flow cytometry analysis used in this study. a-b) Gating strategy used to define the population of CD4+ T cells subsets from spleen (a) and brain (b) in this manuscript.

Supplementary Figure 5: Original blot images corresponding to the Fig. 2b in the manuscript.

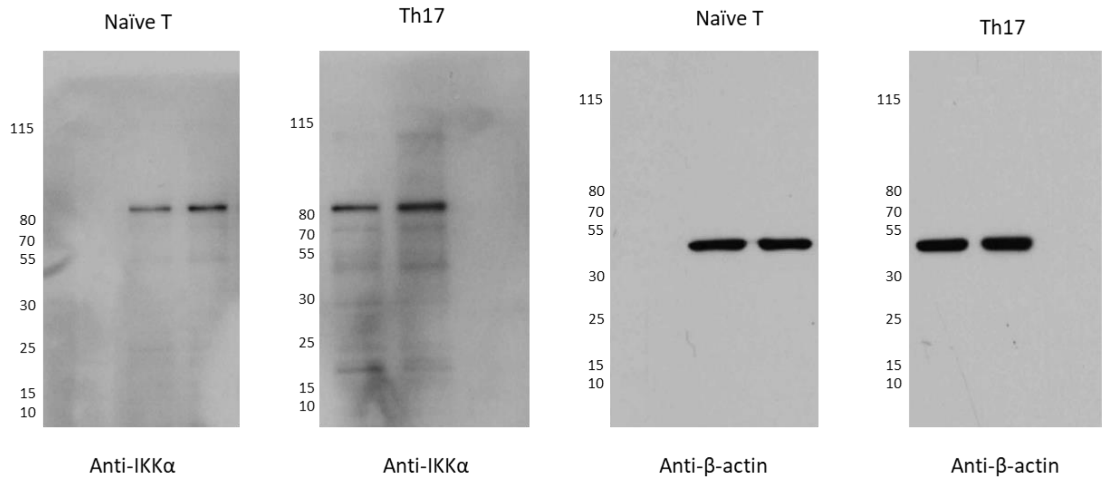

Supplementary Figure 6: Original blot images corresponding to the Fig. 2e in the manuscript.

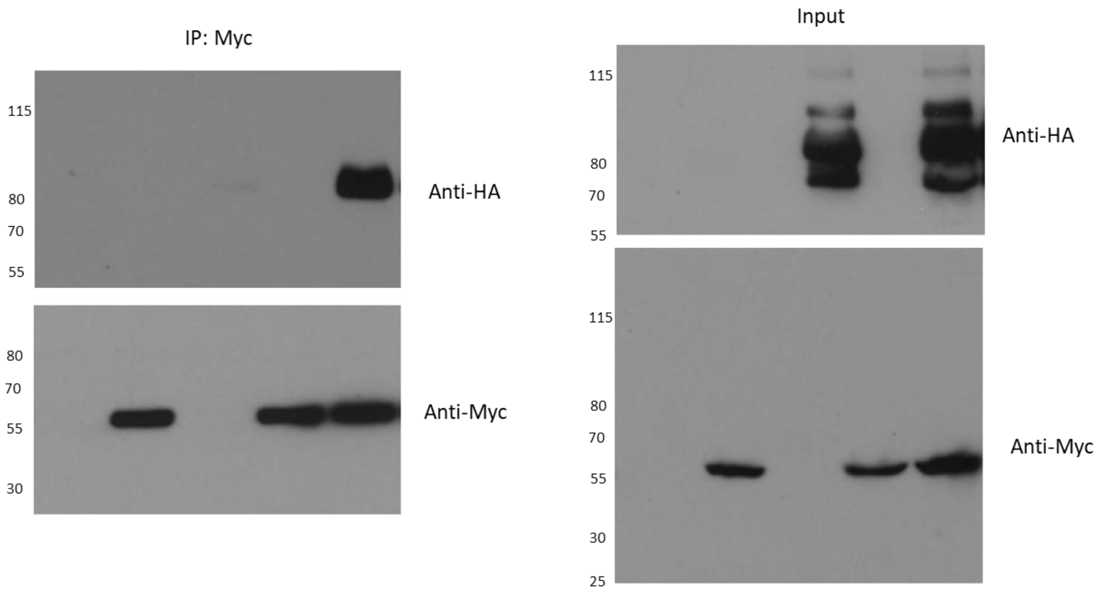

Supplementary Figure 7: Original blot images corresponding to the Fig. 3a in the manuscript.

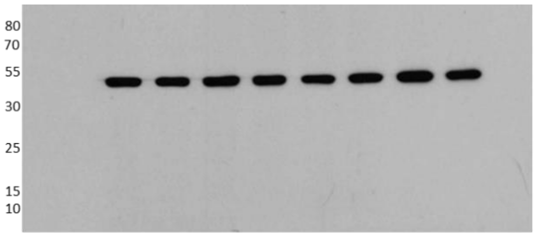

Anti-β-actin

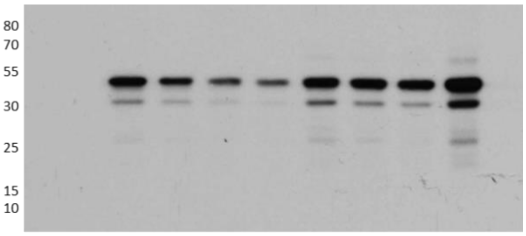

Anti-Foxp3

Supplementary Figure 8: Original blot images corresponding to the Fig. 3b in the manuscript.

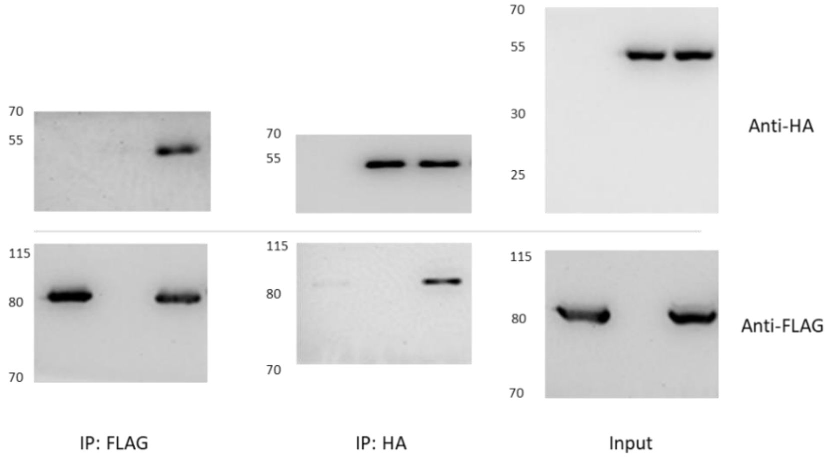

Supplementary Figure 9: Original blot images corresponding to the Fig. 3d in the manuscript.

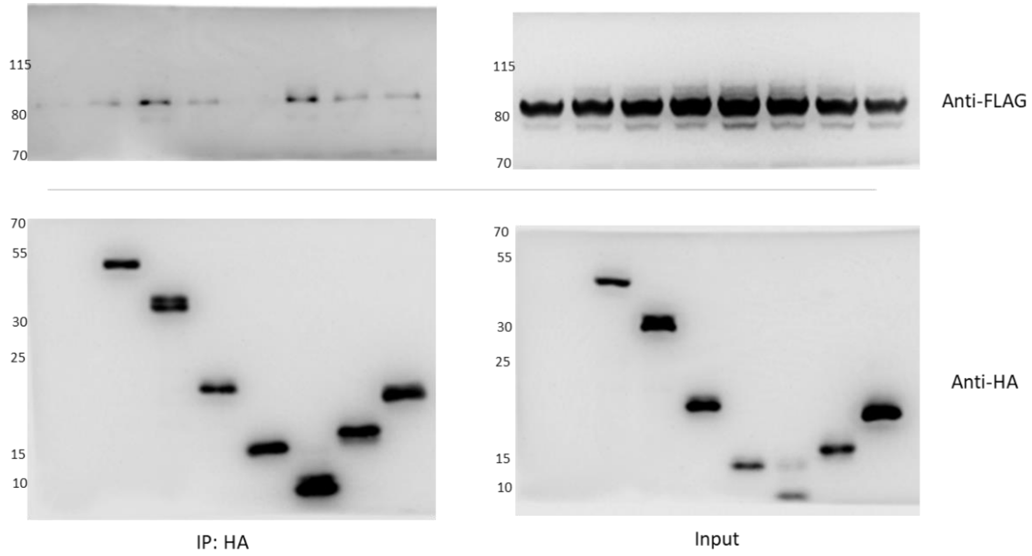

Supplementary Table 1: List of primers used for qRT-PCR.

Supplementary Table 1. Real-time qRT-PCR Primer list

**Gene Name**	Primer sequences	
	Forward	Reverse
Ppar α	5′-CTGCAGAGCAACCATCCAGAT-3′	5′-GTGACCTTCGATTATGCGATCA-3′
Pparγ	5′-ATGCCAAAAATATCCCTGGTTTC-3′	5′-GGAGGCCAGCATGGTGTAGA-3′
Pparδ	5′-GGATTTTAGAGTGGGTGTTTTTTA-3′	5′-CACACCCGATTCCATGTTGAG-3′
IL-17a	5′-CAGACTACCTCAACCGTTCCAC-3′	5′-TCCAGCTTTCCCTCCGCATTGA-3′
IL-17f	5′-AACCAGGGCATTTCTGTCCCAC-3′	5′-GGCATTGATGCAGCCTGAGTGT-3′
Rorc	5′-GTGGAGTTTGCCAAGCGGCTTT-3′	5′-CCTGCACATTCTGACTAGGACG-3′
Rora	5′-CAGAGCAATGCCACCTACTCCT-3′	5′-CTGCTTCTTGGACATCCGACCA-3′
Hif-1α	5′-CCTGCACTGAATCAAGAGGTTGC-3′	5′-CCATCAGAAGGACTTGCTGGCT-3′
Chuk	5′-TCGGAAACCAGCCTCTCAGTGT-3′	5′-CTTCTGGATGCAAATGGTCCTTC-3′
β-actin	5′-GTGAAAAGATGACCCAGATC-3′	5′-CACCGCCTGGATGGCTACGT-3′
