# Supplementary material for: Pparα knockout in mice increases the Th17 development by facilitating the IKKα/RORγt and IKKα/Foxp3 complexes
Source: Commun Biol. 2023 Jul 14;6:721. doi: 10.1038/s42003-023-05104-6 (PMC10349144; doi:10.1038/s42003-023-05104-6)
Supplement: Supplementary file 2 — Description of Additional Supplementary Files [file 42003_2023_5104_MOESM2_ESM.pdf]

Description of Additional Supplementary Files

File name: Supplementary Data 1

Description: The source data behind the graphs in the manuscript.
