# Supplementary material for: Pparα knockout in mice increases the Th17 development by facilitating the IKKα/RORγt and IKKα/Foxp3 complexes
Source: Commun Biol. 2023 Jul 14;6:721. doi: 10.1038/s42003-023-05104-6 (PMC10349144; doi:10.1038/s42003-023-05104-6)
Supplement: Supplementary file 4 — Reporting Summary [file 42003_2023_5104_MOESM4_ESM.pdf]

nature portfolio

Corresponding author(s): COMMSBIO-22-3234B

Last updated by author(s): Jun 26, 2023

Reporting Summary

Nature Portfolio wishes to improve the reproducibility of the work that we publish. This form provides structure for consistency and transparency in reporting. For further information on Nature Portfolio policies, see our Editorial Policies and the Editorial Policy Checklist.

Please do not complete any field with "not applicable" or n/a. Refer to the help text for what text to use if an item is not relevant to your study.
For final submission: please carefully check your responses for accuracy; you will not be able to make changes later.

Statistics

For all statistical analyses, confirm that the following items are present in the figure legend, table legend, main text, or Methods section.

n/a	Confirmed	
☐	☒	The exact sample size (n) for each experimental group/condition, given as a discrete number and unit of measurement
☐	☒	A statement on whether measurements were taken from distinct samples or whether the same sample was measured repeatedly
☐	☒	The statistical test(s) used AND whether they are one- or two-sided<br>*Only common tests should be described solely by name; describe more complex techniques in the Methods section.*
☒	☐	A description of all covariates tested
☒	☐	A description of any assumptions or corrections, such as tests of normality and adjustment for multiple comparisons
☒	☐	A full description of the statistical parameters including central tendency (e.g. means) or other basic estimates (e.g. regression coefficient) AND variation (e.g. standard deviation) or associated estimates of uncertainty (e.g. confidence intervals)
☒	☐	For null hypothesis testing, the test statistic (e.g. F, t, r) with confidence intervals, effect sizes, degrees of freedom and P value noted<br>*Give P values as exact values whenever suitable.*
☒	☐	For Bayesian analysis, information on the choice of priors and Markov chain Monte Carlo settings
☒	☐	For hierarchical and complex designs, identification of the appropriate level for tests and full reporting of outcomes
☒	☐	Estimates of effect sizes (e.g. Cohen's d, Pearson's r), indicating how they were calculated

Our web collection on statistics for biologists contains articles on many of the points above.

Software and code

Policy information about availability of computer code

Data collection	ImageJ (v 1.6.0), Attune NxT (ThermoScietific), FlowJo (v10)
Data analysis	Graphpad prism

For manuscripts utilizing custom algorithms or software that are central to the research but not yet described in published literature, software must be made available to editors and reviewers. We strongly encourage code deposition in a community repository (e.g. GitHub). See the Nature Portfolio guidelines for submitting code & software for further information.

Data

Policy information about availability of data

All manuscripts must include a data availability statement. This statement should provide the following information, where applicable:

- Accession codes, unique identifiers, or web links for publicly available datasets
- A description of any restrictions on data availability
- For clinical datasets or third party data, please ensure that the statement adheres to our policy

All the data generated is original, and described in the manuscript. Raw data are available from the corresponding author upon reasonable request.

Human research participants

Policy information about studies involving human research participants and Sex and Gender in Research.

Reporting on sex and gender	N/A
Population characteristics	N/A
Recruitment	N/A
Ethics oversight	N/A

Note that full information on the approval of the study protocol must also be provided in the manuscript.

Field-specific reporting

Please select the one below that is the best fit for your research. If you are not sure, read the appropriate sections before making your selection.

☒ Life sciences ☐ Behavioural & social sciences ☐ Ecological, evolutionary & environmental sciences

For a reference copy of the document with all sections, see nature.com/documents/nr-reporting-summary-flat.pdf

Life sciences study design

All studies must disclose on these points even when the disclosure is negative.

Sample size	All experiments were preformed 3+ times.
Data exclusions	No data was excluded.
Replication	All in vivo, in vitro, and flow experiments were performed at least 2+ times
Randomization	Littermate mice were randomly grouped into control and treatment groups for all experiments in this study.
Blinding	EAE scoring was done blinded. FACS and molecular analyses by nature required grouping of data and could not use blinding.

Reporting for specific materials, systems and methods

We require information from authors about some types of materials, experimental systems and methods used in many studies. Here, indicate whether each material, system or method listed is relevant to your study. If you are not sure if a list item applies to your research, read the appropriate section before selecting a response.

Materials & experimental systems

n/a	Involved in the study	
☐	☒	Antibodies
☐	☒	Eukaryotic cell lines
☒	☐	Palaeontology and archaeology
☐	☒	Animals and other organisms
☒	☐	Clinical data
☒	☐	Dual use research of concern

Methods

n/a	Involved in the study	
☒	☐	ChIP-seq
☐	☒	Flow cytometry
☒	☐	MRI-based neuroimaging

Antibodies

Antibodies used	IFN-γ PE, IFN-γ APC, IL-13 PE, IL-17 APC (BD Bioscience), IL-2 APC (BD Pharmingen), Foxp3 PE (eBioscience). IKKα (Cell Signaling Technology), β-actin (Proteintech), Foxp3 (Proteintech), Anti-mouse IgG (Cell Signaling Technology) and anti-rabbit IgG (Cell Signaling Technology).
Validation	Antibodies has been validated in multiple papers, and have been used in our lab for long time.

Eukaryotic cell lines

Policy information about cell lines and Sex and Gender in Research

Cell line source(s)	293T cell line commonly used in our lab was bought from ATCC
Authentication	Cells were bought from authentic source. Meanwhile, microscopic examination was conducted to verify the cell shape and cell growth.
Mycoplasma contamination	Cell line was regularly check for Mycoplasma contamination. It was Mycoplasma-free throughout the experiments.
Commonly misidentified lines (See ICLAC register)	N/A

Animals and other research organisms

Policy information about studies involving animals; ARRIVE guidelines recommended for reporting animal research, and Sex and Gender in Research

Laboratory animals	C57/BL6 Ppara KO mice were purchased from the Jackson Laboratory. Stat3 f/f; Cd4-cre mice were previously described. Ppara KO mice were crossed to Stat3 f/f; CD4-cre to generate DKO mice.
Wild animals	N/A
Reporting on sex	N/A
Field-collected samples	N/A
Ethics oversight	All animal experiments complied with the Johns Hopkins Animal Care and Use Policy.

Note that full information on the approval of the study protocol must also be provided in the manuscript.

Flow Cytometry

Plots

Confirm that:

☒ The axis labels state the marker and fluorochrome used (e.g. CD4-FITC).

☒ The axis scales are clearly visible. Include numbers along axes only for bottom left plot of group (a 'group' is an analysis of identical markers).

☒ All plots are contour plots with outliers or pseudocolor plots.

☒ A numerical value for number of cells or percentage (with statistics) is provided.

Methodology

Sample preparation	For extracellular staining, harvested cells were washed and incubated in PBS containing 1% FBS containing the below fluorochrome-conjugated antibodies in a U-bottom 96-well plate. For intracellular cytokine staining, harvested cells were re-challenged in PMA and Ionomycin in the presence of Golgi-Plug (BD Biosciences). After 5 hours of incubation, the cells were fixed/permeabilized (eBioscience) and incubated with antibodies. The following antibodies were used for the flow cytometric assay: IFN-γ PE, IFN-γ APC, IL-13 PE, IL-17 APC (BD Bioscience), IL-2 APC (BD Pharmingen), Foxp3 PE (eBioscience).
Instrument	Attune NxT Flow Cytometer was used.
Software	Flowjo_ V10 was used for data analysis.
Cell population abundance	95% cells were purified as determined by flow cytometry.
Gating strategy	Brain mononuclear cells or spleen sells were gated for lymphocytes by FSC, SSC, doublets were discriminated, and live cells were analyzed for CD4+ expression. CD4+ T cells were further analyzed for IFN-γ+, IL-13+, IL-17+, IL-2+, and Foxp3+ cells.

☒ Tick this box to confirm that a figure exemplifying the gating strategy is provided in the Supplementary Information.

This checklist template is licensed under a Creative Commons Attribution 4.0 International License, which permits use, sharing, adaptation, distribution and reproduction in any medium or format, as long as you give appropriate credit to the original author(s) and the source, provide a link to the Creative Commons license, and indicate if changes were made. The images or other third party material in this article are included in the article's Creative Commons license, unless indicated otherwise in a credit line to the material. If material is not included in the article's Creative Commons license and your intended use is not permitted by statutory regulation or exceeds the permitted use, you will need to obtain permission directly from the copyright holder. To view a copy of this license, visit http://creativecommons.org/licenses/by/4.0/
